# DISRUPTIVE IRRITABILITY & FAMILY DYSFUNCTION CORRELATION: ANALYSIS THROUGH FAMILY DRAWINGS

**DOI:** 10.1192/j.eurpsy.2023.1497

**Published:** 2023-07-19

**Authors:** A. Vargas Castro

**Affiliations:** 1 Child & Adolescent Psychiatry, Hôpital de la Pitié Salpêtrière, Paris; 2 Child & Adolescent Psychiatry, Centre Hospitalier de Versailles, Versailles, France

## Abstract

**Introduction:**

Drawing represents mainly a motor activity of expression. Drawing represents a form of non-verbal language that is very important both cognitively and affectively. Therefore, it allows to hypothesise and evaluate the degree of neurodevelopment of individuals as well as their level of interaction with the environment.

Family sketches can be evaluated in a projective, neurocognitive and affective way to provide insights on the attachment system, degree of bonding, communication, social and affect interaction as well as difficulties or problems that have motivated emergency consultation.

**Objectives:**

This study evaluate the possible correlation between family dysfunction and irritability as cause of request of consultation in an emergency department of mental health in child & adolescents through the analysis of family drawings.

**Methods:**

This is a retrospective, observational study of correlation between the reasons of emergency consultations, dysfunctional irritability and family difficulties represented through family drawing. It is based on a randomised sample of 30 reports of emergency appointments of children between 8 to 13 years old that have been examined in the Child & Adolescent Psychiatry Emergency Department at the Pitié Salpêtrière Hospital during two years for Emotional or Irritability dysfunction.

An adaptation of both Goodenough-Harris Drawing projective test and Corman test were used to evaluate findings from family drawings as well as neurocognitive parameters of drawing technics, sociodemographic dates, cognitive level and family dysfunction.

**Results:**

The degree of cohesion, identification and devaluation of adult figures have been important elements of interpretation in irritability dysfunction and family drawings.

**Conclusions:**

The family environment could be a factor in the interpretation of chronic irritability and its manifestations on the child’s family drawings establish a clear correlation.

The adapted assessment of the family drawing could be an important tool in the nosological exploration of children’s mental health in emergency, especially on relational systemic representation and symbolization.

**Disclosure of Interest:**

None Declared

